# Development of a smartphone virtual reality game to support the radiation therapy of children and adolescents in proton centers

**DOI:** 10.3389/fped.2023.1163022

**Published:** 2023-06-20

**Authors:** Leonardo Schenck, Christian Bäumer, Björn Ross, Gabriele Schäfer, Nicole Stember, Heike Thomas, Stefan Stieglitz, Beate Timmermann

**Affiliations:** ^1^ West German Proton Therapy Centre Essen (WPE), Essen, Germany; ^2^ Department of Computer Science and Applied Cognitive Science, University of Duisburg-Essen, Essen, Germany; ^3^ West German Cancer Center (WTZ), University Hospital Essen, Essen, Germany; ^4^ German Cancer Consortium (DKTK), Essen, Germany; ^5^ Department of Physics, TU Dortmund University, Dortmund, Germany; ^6^ Department of Particle Therapy, University Hospital Essen, Essen, Germany

**Keywords:** proton therapy, virtual reality, pediatric cancer patients, patient immobilization, serious games, phosphenes

## Abstract

**Introduction:**

For most patients, cancer therapy with radiation is a new experience coming with many unknown challenges. This can be stressful, particularly for children and adolescents. With the aim of reducing this stress and anxiety, a virtual-reality (VR) game, which can be used by patients prior to treatment, was developed and evaluated in a proton therapy center.

**Methods:**

The specifications were derived from literature and from interviews with medical staff and patients. The gantry including the sound of its moving components and the sound of the interlock and safety system were identified as the main features relevant for preparation of a radiation course. Potential implementation difficulties were identified in a literature study and regarded in the design. Within the VR game, patients could interact with modeled equipment of the treatment room and hear the reportedly stress-inducing sounds in a stress-free environment prior to the treatment. The VR game was evaluated in a second series of interviews with patients.

**Results and Discussion:**

This exploratory study demonstrated the specification, implementation and safe application of a VR game dedicated to young proton therapy patients. Initial anecdotal evidence suggested that the VR gaming experience was well received and found to be helpful when preparing young patients for radiation therapy.

## 1. Introduction

Proton therapy makes use of the characteristic depth dose distribution, which features a limited entrance dose, a well defined peak dose and a steep dose fall-off. Compared to conventional radiation therapy with hard x-rays, the normal tissue can be better spared while delivering the same dose to the target volume. In general, children are very sensitive to radiation. This concerns, e.g., the risk for later cognitive impairment. Thus, younger patients in particular may potentially benefit from proton therapy due to a reduced risk of relevant side effects and a lower probability of secondary malignancies. Therefore, there is great interest in proton beam therapy of pediatric patients. For instance, the median age of the entire patient cohort at the West German Proton Therapy Centre Essen (WPE) is 15.5 years and more than half of all patients are younger than 18 years. Initial experience from clinical cohorts at WPE and elsewhere has already been published (1–8).

Especially for younger patients, the new and unknown environment can be stressful and frightning. During the treatment sessions, which typically lasts 20 min but can take up to 1 h, the patient has to lie still. Very young patients (3–6 years of age) are predominantly unable to cooperate and may require general anaesthesia for the radiotherapy sessions to ensure immobilization. Sometimes even older patients have difficulties to comply consciously with positioning procedures during radiation therapy. However, sedation can be logistically demanding and physically and emotionally stressful for the patients and the parents. To avoid general anaesthesia and to improve the overall quality of life for the patient during the treatment course, several supportive measures and mental aids are provided throughout the therapy. For instance, the medical team and the psycho-oncology team provide in-person consultations during the outpatient stay at the proton center. In addition, comics serve as educational tool for radiotherapy training of young patients. Some patients may listen to music or audiobooks within the treatment rooms to distract themselves. However, the environment still has an impact on the emotions due to the effects of the unknown environment, noise, huge machines, and distinct perceptions during the treatment, which are difficult to prepare for.

It has been suggested, that the immobilization of younger patients could potentially be supported by serious games and their Virtual Reality (VR) implementation (9, 10). A new branch of preparation and habituation has emerged from the video-games sector under the name “serious games.” Whilst “gamification” describes the use of game-elements in a non-game-environment for motivation, the user of serious games is considered as a player rather than a learner, i.e., serious games are actual games. It has not been defined whether the focus is on the educational or the game elements (11). They can be used to prepare medical doctors in crises, to learn languages faster or to understand more complex processes (12). In one popular serious game, “re-mission”, which was followed up by the game “re-mission 2”, the player can fight against and defeat cancer cells by shooting them. Patients, who played this game, had more knowledge about cancer and showed more health-oriented behavior and cooperation throughout the therapy (13).

VR is a medium, which allows for a high level of immersion into the game. It facilitates a feeling of being in the given world as, e.g., books, smartphone games, games on personal computers etc. According to the literature, VR is associated with a potentially higher acceptance of the game's elements. For patients with severe burns, a game called “Snow-World”, exposed the player to a snowy reality as distraction (14). Here, it could be demonstrated, that the patients experienced less pain compared to patients, who did not use the VR game. In a similar environment during surgery, the VR led to less movements in the real world, simplifying the process for the doctors (15). VR has also been used in museums to please younger audiences and grant more immersion (16). Hundert et al. (17) evaluated VR-based distraction to reduce procedural pain during subcutaneous port access in pediatric and adolescent cancer patients. Ricciardi and De Paolis (18) argued that VR could be useful for the training of new medical doctors in specialized surgeries, e.g., after earthquakes, when many patients had wounds but doctors were untrained. Here the surgeries conducted after VR training were significantly better than the ones performed without training. In addition, the results were almost as good as those of a third group of doctors with training under real conditions or when training with animals. Regarding cancer therapy with radiation, the Virtual Environment for Radiotherapy Treatment (“VERT”, Vertual, Hull/UK) has been developed to train healthcare professionals (19). This software has been adopted for patient education in a number of studies [Grilo et al. (20) and references therein]. Under the acronym VR-RLX a method has been developed to prepare children for their MRI-sessions (21). Another study found lower anxiety and distress scores for pediatric patients, who were prepared with VR-based education to chest radiography (22).

**Figure 1:**
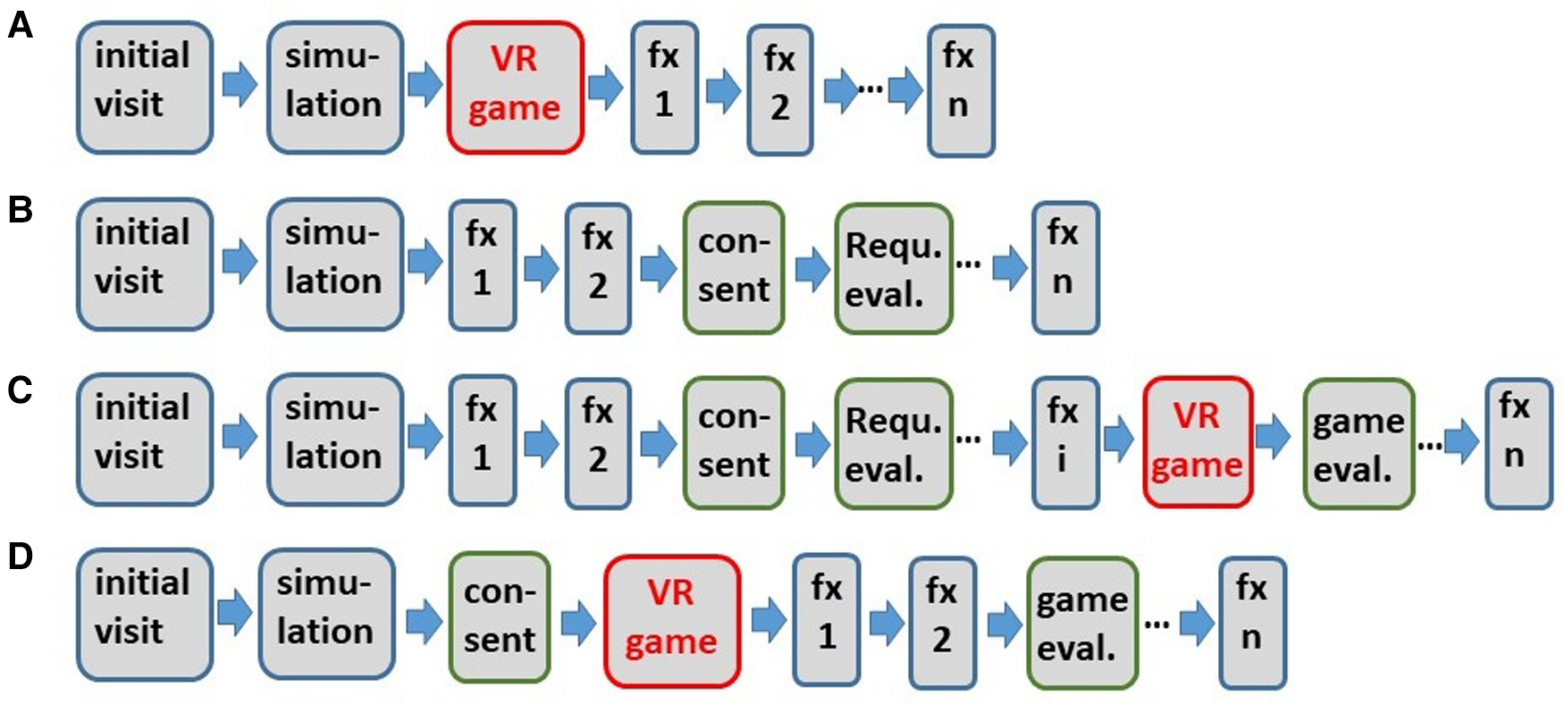
Flow chart of the use of the VR game in a clinical environment (**A**) and involvement of the patients in the implementation phase of the VR game (**B–D**): patient group 1 (**B**), three patients, which are in patient group 1 and group 2 (**C**) patient group 2 (**D**). Blue bordered blocks refer to steps of the conventional clinical workflow including the simulation and the treatment fractions “fx”. Simulation refers to the adaption of immobilization devices, e.g., head masks and vacuum cushions, and an x-ray CT scan. The number *n* of fractions fx is typically 30–33. “Requ. eval.” refers to the requirement evaluation with patient interviews.

Thus, it is expected that a VR application with game elements (“VR game”) could address various aspects of preparation for radiation therapy that need to be identified and defined by the individual radiation oncology institutions. The purpose of the current project was to create a VR-based serious game for a proton therapy center with a special focus on the treatment of children with cancer. The VR application shall be used by young patients to support the preparation of the treatment sessions in a proton gantry room. Figure 1A visualizes the foreseen incorporation of the VR game into the clinical workflow. Furthermore, the current study aimed to learn how a VR game can be integrated under the specific requirements of a radiation therapy institution and how it is perceived by staff and patients. Several criteria must be met: (a) potential participants should have a high level of interest in using the VR game, (b) a majority of participants must recognize elements of the treatment environment from their depiction in the VR game, (c) the usage must be joyful, focused, and must not disturb the clinical procedure at the proton therapy center.

## 2. Methods and study design

The project was approved by the Ethics Board of the medical faculty of the University Duisburg-Essen under application 17-7821-BO (ID DRKS00013601 of the German Clinical Trials Register) prior to the start of the evaluations with patients and the corresponding interviews and prior to the software development. Patients between 7 and 16 years of age who were irradiated at the WPE were eligible for the study, regardless of the type of tumor disease. Patients with visual impairment, double vision or a history of dizziness, or seizures were excluded from the study.

The requirement document for the VR game (Section 3) captured the input from health care professionals (Section 3.1) as a first step. Interviews with the clinical staff of the clinic of particle therapy in the WPE were conducted to assess the current procedures for the treatment of young patients. These conversations lasted about 20 min, were open-ended, and the questions varied among the interviewees. In particular, it was considered that the VR game should be smoothly embedded into the existing procedures for preparing young patients for proton therapy (Section 3.2). Subsequently, five patients between the ages of 9 and 16 years were interviewed (Section 3.3, Figure 1B and group number 1 in Table 1) to figure out how they experienced their first treatment, what they would expect from a VR game and to learn about their feelings and perceptions in general. The interviews were semi-structured and qualitatively followed Longhurst (23). They included prepared questions, but were conducted freely, and most of the interview time was left open for the participant to speak their mind. All interviews in this study were conducted in German. The illustrative quotes from patients (Section 3.3 and Section 5) are literal translations of the recorded feedback. The requirements of staff and patients were evaluated and combined (Section 3.4). Supplementary information for the requirements was taken from the literature and current development standards (Section 3.5). The requirement analysis and the development process were based on the design science approach according to Hevner et al. (24).

**Table 1:** Description of the groups [number (“#”) 1 and number 2] of the patients interviewed.

#	Patient group	Cohort	Age	Comment
1	Requirement evaluation	3 female, 2 male	9–16	
2	VR game evaluation	4 female, 1 male	8–13	3 patients identical with first group

**Figure 2:**
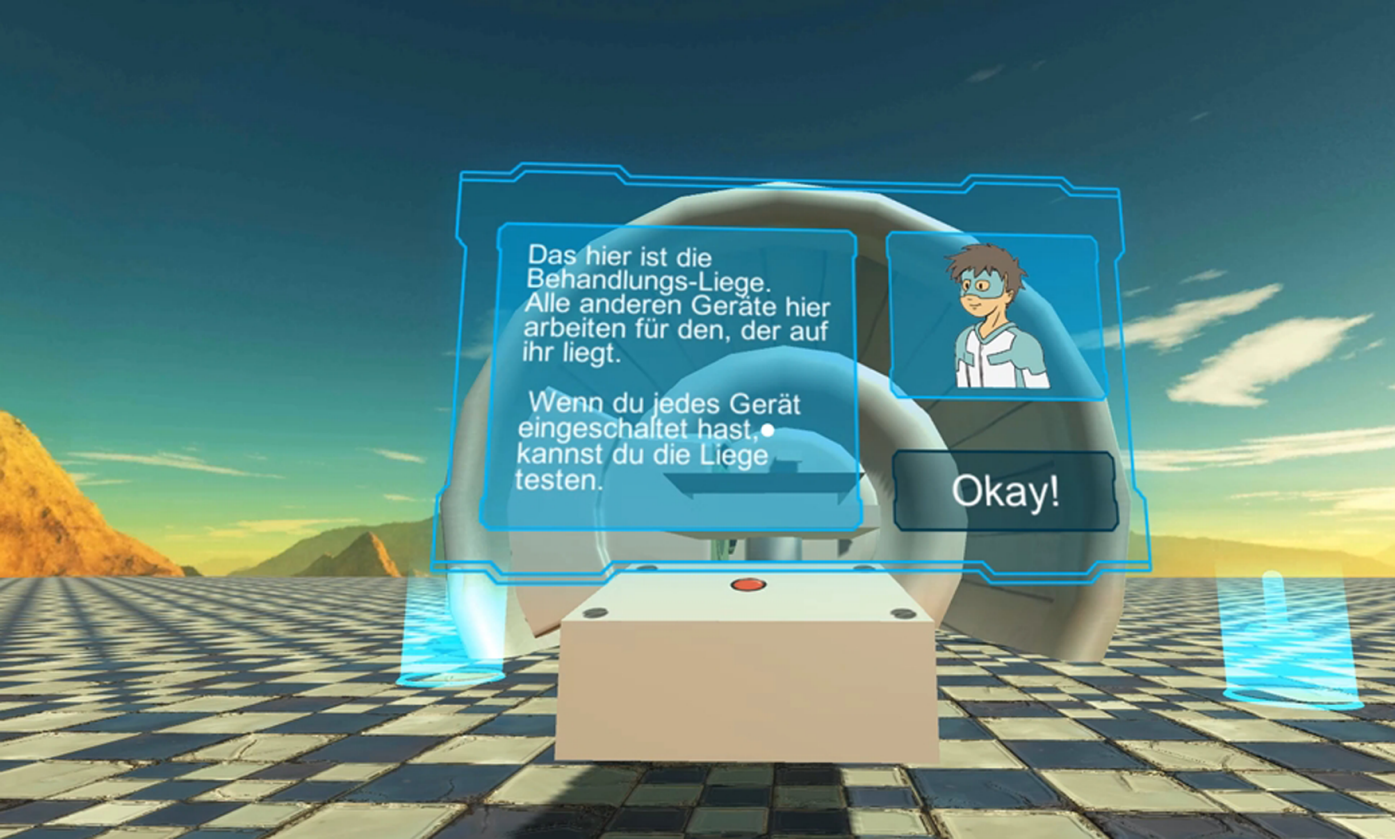
Exemplary screenshot of the VR game (German version). The text message in the left sub-window is “This is the treatment couch. Every other device you see will work for the benefit of the one lying here. Once you have activated all the other devices, you can test the treatment couch for yourself!”

The realization of the VR game (Section 4) utilized a standard smartphone with a standard operating system together with a standard VR head-mounted display (“goggle”). Furthermore, the implementation of the VR game was conducted with a widely used software development environment. Figure 2 shows an exemplary screenshot of the developed VR game. At the start of the application, a dialog, which is visible in the foreground with the protagonist and text, and the gantry with the patient couch are shown.

After the implementation phase, the potential impact of the VR game was evaluated in a qualitative study with a single-arm design. In this context, a second group of patients was selected to test the game before their first treatment session (Section 5). This group was extended by patients who were on treatment and had already been interviewed for the requirement analysis. As a result, the final group (group number 2 in Table 1 and Figure 1C) consisted of two new patients and three who were already under treatment and who were also part of the first group. Six to ten days prior to the first treatment day, they played through the game and were asked a few questions about aspects of the game (Figure 1D). On their second treatment day, they were interviewed a second time to evaluate their perception of their treatment.

While playing the VR game, the patients were asked to describe what they saw, if they were confused or what they found awesome. This feedback was documented by the interviewer. After the playing time, the patients were asked about specific concepts within the VR game such as the sound, the realization of movements and the perception of the mini-games as well as the overall fun and the intuitiveness. After 10–20 min of VR game usage, the interview lasted another 5 min. The post-treatment interviews took about 10 min.

Eventually, the results of the study are discussed (Section 6).

## 3. Requirement analysis

### 3.1. Requirements addressed by medical staff in general

**Figure 3:**
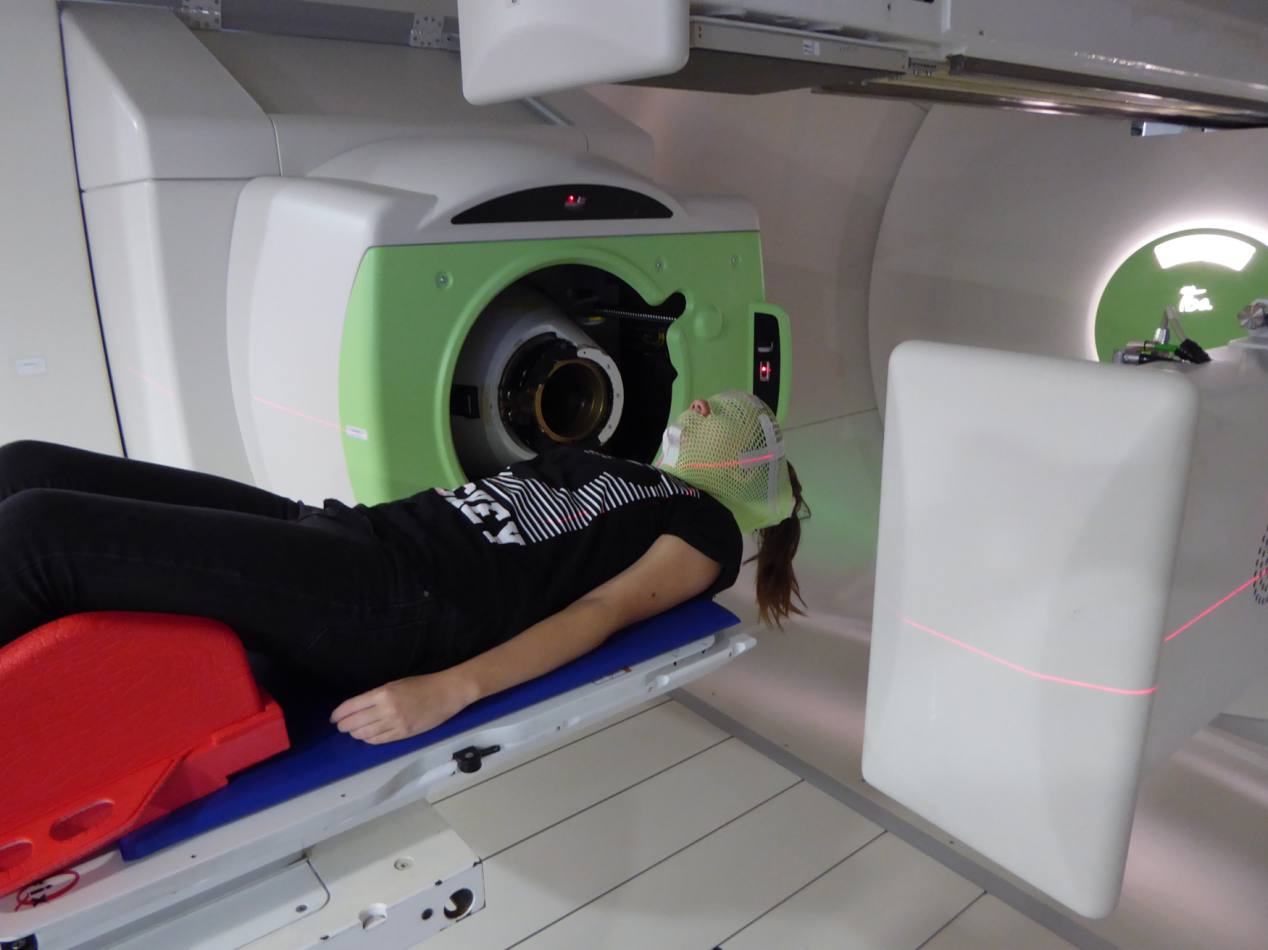
Photo of the inner part of the gantry with proton nozzle (left), treatment couch, extended flat x-ray panels, and a patient with head immobilizing mask (light green color). The red LASER light for initial patient positioning is turned on.

From the staff conversations it was taken that a VR game for treatment preparation would be appreciated. One of the most frequently mentioned requirements were the sound and the visualization of the machines, both of which are perfect for inclusion in a VR game. “Machine” refers to a treatment room of the proton therapy center of the ProteusPlus type (IBA PT, Lovain-la-Neuve, Belgium), which is equipped with a ± 180° gantry and a universal nozzle providing three types of proton delivery modes, a dedicated eye-line and a fixed-beam treatment room. The machine components visible in the treatment room belong to the gantry vault, i.e., a large rotating machine (4 m inner diameter) on which the treatment head and the x-ray panels for position verification are mounted. The treatment couch is located in the center of the gantry, which rotates around the patient lying on the couch (Figure 3). In addition, there are other pieces of equipment, but these seemed to be of minor concern to the patients. The sounds consist of a background noise generated by the vacuum pumps, and additional sounds generated by the movement of the equipment, which are not necessarily signaled or announced in advance. The movement of the treatment couch, the rotation of the gantry and the extracting of x-ray panels all have their own distinct sounds.

Another wish expressed by the WPEs staff was a stress-free virtual environment, which is free of undesirable perceptions. Furthermore, the content of the game should relate to aspects associated with the WPE environment in real life and no information presented should be false or misleading.

### 3.2. Requirements with regard to treatment preparation

The preparation for treatment at WPE starts with an initial visit. Herein, the patients and their relatives are introduced to the treatment techniques thereby receiving a lot of information, e.g., logistical information for the parents. Age-appropriate preparation of the children is done, too. In addition, materials such as comics with, e.g., the booklet on “Radio Robby” are provided for the patients. “Radio Robby” is a comic created and owned by the “Deutsche Kinderkrebsstiftung” (German Childhood Cancer Foundation) in which the protagonist “Radio Robby” fights against cancer cells. Recently, WPE has created a new WPE-hero tailored to the specific, local environment. In particular, a comic book with the title “Proton Mission” has been created with the WPE-hero as the main character (38).

The next day after the initial visit, preparations for the treatment planning will follow. After that, the patient may return home for about 10 days until the proton therapy starts. From the start of proton therapy until its completion, the patient is typically irradiated once a day, five times per week for up to 7 weeks.

### 3.3. Requirements evaluated by patient interviews

The patient interviews were conducted within the first 3 days of treatment, when the memory of the new experience was still fresh. The prepared questions are documented in the Supplementary Material. The children (group number 1 in Table 1) reported that the noises were the most prominent aspect of their perception during therapy, although they also mentioned that they were well announced in advance and never came as a surprise. The machines were described as “like something out of Doctor Who” and “from outer space” or “out of this world”. Another aspect of the treatment, reported by four of the five participants, was a distinct smell like “fire”, “gasoline” or “smoke”. Acording to the medical doctors, this odor is reported by many patients, especially young ones, upon start of the irradiation. It might stem from ozone, which is produced by radiation-induced chemical reactions with air. The odor might also be explained by radiation-induced olfactory illusions, which are evoked in the brain (26, 27).

Most of the interviewees described the immobilization face mask, which keeps their head in a reliable, well defined position during the therapy, as “annoying” and “very tight”, with one patient still having the mask's grid pressed into her skin at the time of the interview. Two of the five interviewees mentioned phosphenes (26, 27), i.e., anomalous visual perceptions. They were perceived as blue lightnings appearing in their field of vision during the irradiation. These visual illusions might be linked to the olfactory illusions mentioned above (27).

None of the patients interviewed had to be sedated at their first treatment. When asked to name the predominant impression of the treatment room, everyone mentioned the inner part of the gantry shown in Figure 3, the proton nozzle that is the treatment head, and the treatment couch, whilst no one mentioned anything else. Some of the children had used VR before and all of them used phones and technology on a regular basis. They were all happy and satisfied with the preparation they had. However, some of the patients interviewed also stated, that the treatment room looked very different from what they had expected and that the treatment was still something completely new to them. Four of the five interviewees answered the question “what would you tell yourself before your first treatment?” with “Be less stressed and everything will be fine”, with one also adding “and it stinks.” In general, the patients who were very aware of what was going to happen seemed to be less stressed.

### 3.4. Interpretation of the requirements addressed by literature, medical staff and patients

From the literature, the interviewed patients, the doctors and the medical staff, several requirements were gathered. According to the WPE's representatives, one of the main aspects leading to acute stress, was the “fear of the unknown”, which is understood as a fundamental human emotion (28). In order to reduce or, at best, eliminate this fear, the patient should get more information, experience about the procedures and have a higher level of understanding. This information should always be clear, honest and easy to understand. The virtual environment should be presented through models of the real treatment environment, especially the gantry, the proton nozzle and the treatment couch. These models should be accurate enough to be recognizable, but not too accurate, as this could lead to a phenomenon called the “uncanny valley”, which is described in more detail in the next section. To enhance the immersion, the models should be designed similar and coherent to each other. Additionally, the player should be able to hear the sounds which can be produced by each machine. Furthermore, the other sounds and color schemes displayed in the application should, where possible, reference those perceived in the WPE. The in-game-environment should be stress-free, and the players’ real-world environment should be safe and conducive to concentration. While playing the VR game, the player cannot see the outside world and, thus, someone else needs to be around. The odor is hard to address with VR and, thus, can only be dealt with outside of the VR game. The phosphenes seen by some patients could potentially be integrated. However, we decided to not include this feature in our first implementation, because such a feature could induce additional anxiety before the first fraction. Lying down should be an integral part of the experience in order to demonstrate that is not only harmless but also helpful.

### 3.5. Requirements from literature and technical requirements

Additional requirements were extracted from the literature. To structure the development, Hevner's development framework for information systems was used (24). It specifies that the development of new information systems should be based on two research paths—the “behavioral” and the “design” path. While the behavioral path deals with an existing knowledge foundation, the design path involves the creation of new aspects and additions to the knowledge base. The goal of the first development was to establish a potential solution and to add tested artefacts (partial solutions) to the knowledge base for further development. As the game was to be used in a clinical setting, it was classified as “health software” according to IEC82304-1. In this frame, the target groups characteristics, desired outcomes and potential risks were documented and a software life-cycle process established.

To counteract motion sickness, which is a phenomenon occurring when the perceived motion does not match the actual motion, the in-game camera should move at the same speed as the player's head and without delay (29). Furthermore, only stationary movements should be allowed, i.e., forward movements should only be allowed via teleportation and not by standing up and walking. This is especially important considering the possible physical limitations, morbidities, or radio(chemo-)therapy toxicities of the patient cohort under consideration. During the use of the VR game, the player shall be mainly seated on a rotating chair. Additional hardware-requirements such as screen resolution, display width and required sensors were considered in the hardware selection.

For the content of the game, where applicable, seven gaming-principals should be included. These were formulated by Houser and DeLoach (30) to describe what is needed to make a game enjoyable. The seven principals are: “interactivity, feedback, goals, motivation, challenge, engagement und concentration.” Each of them should be implemented through mini-games, high scores, motivational text and other means. Concentration is the most important aspect of learning to exist within a serious game. To further increase motivation and a positive emotions during the game, the player should experience a “flow” in his actions (31). The difficulty should increase over time, so a feeling of progression ideally comes with an experience of flow. There shouldn't be any disruptions during the use of the VR game and the patient should not experience a moment of not knowing what to do next.

The software shall run on a commercial, cardboard compatible smartphone operated either with Android (Open Handset Alliance, Mountain View/USA) or iOS (Apple Inc., Cupertino/USA). The smartphone shall be mounted in Google-VR goggles. Table 2 gives an overview of the requirements of the VR game.

**Table 2:** Requirements of the VR game.

Source/rationale	General and environment requirements	Content requirements
Interviews with healthcare professionals of the proton center	- No nausea shall be caused by the VR game - Information shall be correct, but not stress or fear inducing - Incorporation of the study into the therapy workflow: VR game use/patient interviews after the initial visit/first treatment fractions	- Visualization of proton treatment machine; - Noises associated with the therapy machine/workflow shall be recognizable - The color scheme shall resemble the one of the therapy center
General considerations	- Use of VR game in rotating chair in a supervised quiet room (hospital environment, prevent accidents) - Simple understandable texts (target group)	
Interviews with patients		- Visual representation of treatment machine - Noises associated to treatment shall be recognizable - Incorporate superhero as protagonist, e.g., Radio Robby (from Deutsche Kinderkrebsstiftung)
Literature	- Adhere to the 7 gaming principles of Houser and DeLoach (30) - Flow experience regarding user actions (31) - Recognizable machines, but no realism	

## 4. Development of the smartphone-based VR game

### 4.1. Software tools and software design

Unity (Unity Technologies, San Francisco/USA) and Visual Studio (Microsoft, Redmond/USA) were chosen as the integrated development environment. Models were created in 3dsMax (Autodesk, San Rafael/USA) and Photoshop (Adobe, San Jose/USA) and sounds were either recorded at the WPE with a Behringer Microphone (Willich/Germany) and the software Audacity (Audacity, https://audacityteam.org) or were downloaded from free sound databases.

**Figure 4:**
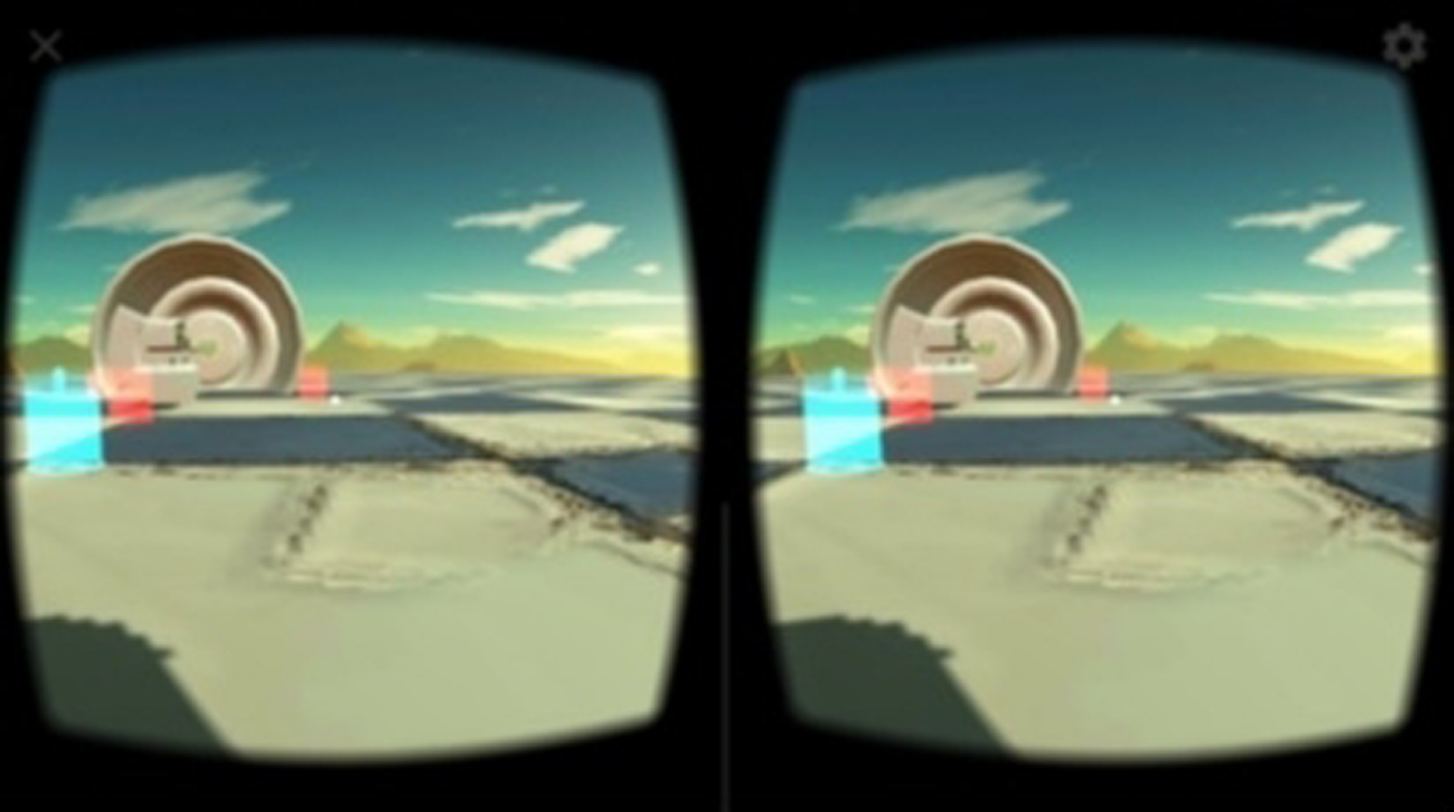
Split view of the VR game on the smartphone (screenshot).

There are many factors to consider when developing 3D-Games. For instance, games are expected to incorporate physical attributes, object rendering, gravity and sound. Because these complex components are needed in more than just one game, games are usually built with a game-engine—a bundle of functions that the programmer can access via application programming interfaces, which are usually packaged in a software development kit (SDK). Unity comes with a built-in game-engine and support for multiple platforms, including the Android and Apple iOS operating systems, making it the ideal choice for this project. The visual editor makes it easy to start developing and see results. Visual Studio is easily integrable into Unity and supports the used coding-language C#. With Unity, the programmer is able to place objects in the world and give them functions. These objects were built with Photoshop and 3dsMax. Git was used as the version control system. Bitbucket was used to communicate the code between the different contributors. To develop the game as a VR game for the smartphone, the Google VR SDK was used. It displays the scene for each eye, splits the screen in half (Figure 4) and facilitates to rotate the in-game camera dependent on the gyroscope data of the smartphone, which corresponds the player's head movements.

**Figure 5:**
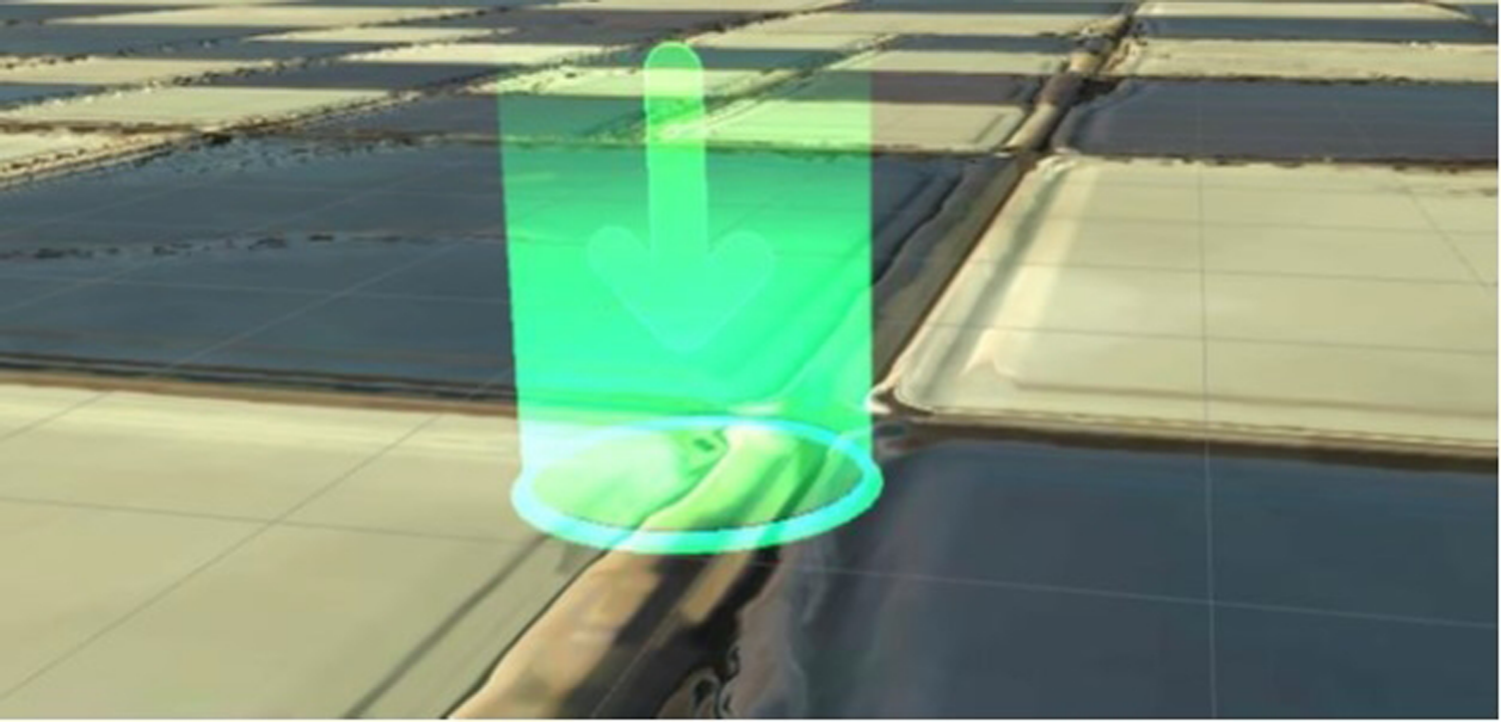
Teleport point in the VR game.

**Figure 6:**
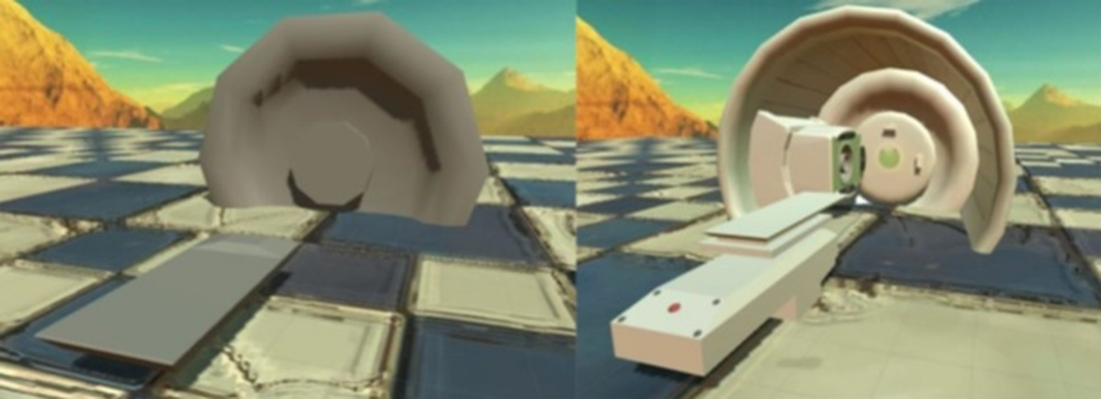
Development iterations of the gantry including nozzle and treatment couch (screenshot).

**Figure 7:**
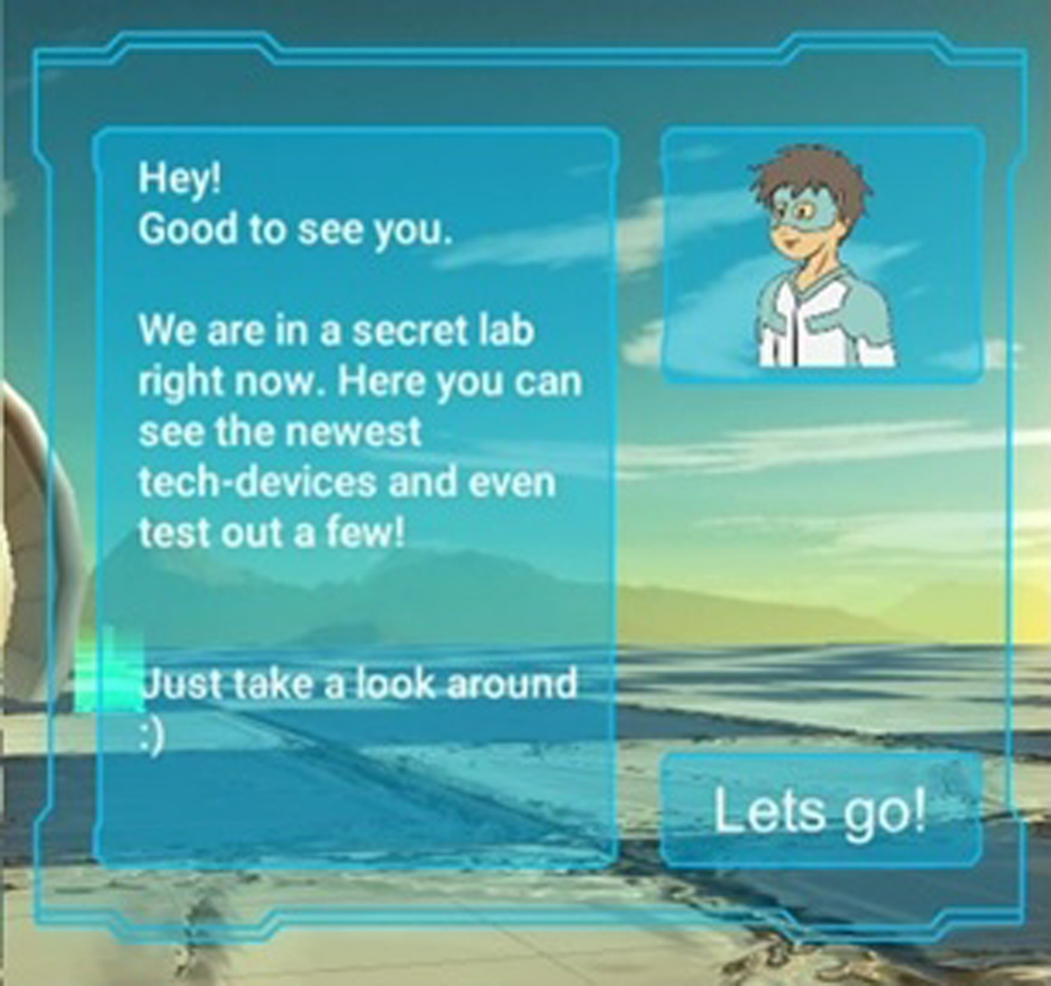
Screenshot of the user interface with the new WPE-hero (top right sub-window).

**Figure 8:**
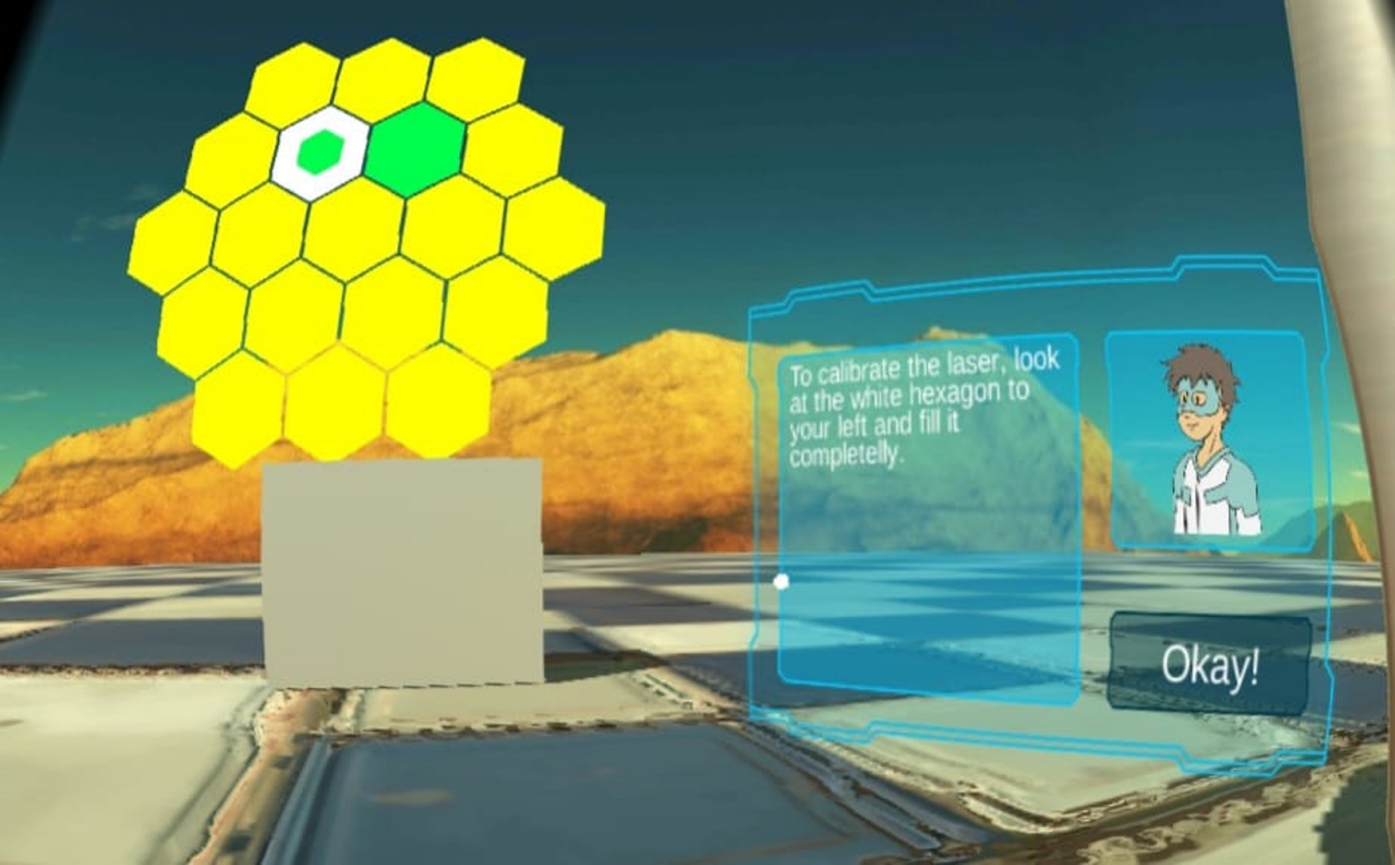
Sight during the last mini-game (screenshot).

Throughout the development process, the software was iterated and improved through testing and feedback from clinical staff. Texts, placement of objects and the pace of the mini-games were adapted to these comments. During the later evaluation process with patients, the VR game was developed further as well, though key concepts remained untouched. After the VR game was evaluated, the development continued, and additional changes were incorporated. Performance in terms of a smooth, uninterrupted motion experience was a high priority in the software design. In order to facilitate this performance on a wide range of smartphone models, low-resolution textures and low-poly models were used in Unity. This allowed for a high processing speed while maintaining a sufficient image quality with a VR goggle. However, the limited image resolution is discernible in the monocular display of the VR game screenshots in Figures 2, 4–8.

### 4.2. Game contents

Since the player cannot use hands or legs or anything but head movement, interactions with, e.g., buttons become tricky. A system called “gaze and wait” was implemented for the game. By looking at an interactable object for a certain amount of time, the player can start the interaction. To communicate potential interactions to the player, a small dot is displayed in the middle of the player's view. When the player looks at an interactable object, the dot grows to a small circle and a filling bar appears below it. Once the bar is filled, the interaction starts.

To facilitate the player moving through the virtual environment, teleport markers were placed at each of the three objects, (Figure 5). By looking at them, a sound, the described visuals and additionally the markers changing their color from blue to green, communicate the process of teleporting. When the three seconds are up and the filling bar is full, the player is moved to the new location and can discover new functionalities. If the player is currently engaged in another interaction, the markers will be disabled, turn red, and the arrow will change to a lock.

For the design of the virtual environment a rather free approach was implemented by placing the user in an open landscape of mountains (Figures 4, 6). A room would require a light source, more complex shadows, more resources on the device and more time to model. Any intermediate test version with a room felt oppressive, whereas the free world did not compromise the perception of the machines and created a better feeling overall. The machine models are white and although they do resemble the real ones, they have a more cartoonish approach to them (Figure 6). A photorealistic look would not be achievable, and getting as close as possible would change the feeling of resemblance to an aversion caused by something feeling wrong. This concept is known as the uncanny valley: if it is impossible to achieve the exact same look, then it is advantageous to keep a distance with less detailed modeling (32). It usually refers to living objects rather than inanimate ones, but still the complexity of the models should remain at a low level. The goal was to make the devices recognizable without reaching the uncanny valley.

To communicate text, a user interface (UI) was included (Figures 2, 7). An object attached to the player's view reduces the field of view and is therefore not a good option for VR. Instead, UIs should be placed in the world, which also is the case in this game. The text is always displayed on the left in a window that is large enough. Possible interactions like buttons are in the lower right and in the upper right there is a picture of the game's hero. This anchor person guides the player through the game and gives instructions. In Germany, Radio Robby (Section 3.2) could be used. Radio Robby was used in the first test version of the VR game, However, since the WPE designed a new, more modern and a slightly older hero than Radio Robby, we replaced Radio Robby with the new one, i.e., the WPE-hero. To integrate the UIs into the world, they were made transparent and in a low energy color.

At the start of the game, the object in the middle—the treatment couch (Figure 6)—allows the player to lie down after making certain preparations. Until this is done, the UI next to the couch refers the player to the other two machine components on the left and right. The teleporter (see also Figure 4) to the right belongs to the gantry, which can be activated to show an animation of the gantry turning, accompanied by the corresponding sounds. At the couch's left hand side, the proton nozzle (Figure 5) can be activated. When activated by the player, a two-level mini-game is started in which the player must fill several hexagons to “calibrate the nozzle” (Figure 8). Once these two interactions are completed, the player can lie down in the center of the application. To do so, the player must take off the goggles and lie down on a real couch. Once the VR goggles are back on, a window appears with a marker right in the middle. To the right, a UI explains, how—while looking in the window's center—the player can help the game's hero. On the left, another cluster of hexagons is automatically filled by the hero. If the player is looking away from the center, the filling process slows down. By not moving the head and focusing the gaze, the player can support the progress of the game as well as the treatment. When the cluster is completely colored, the VR game is finished, and the player can take off the VR goggles.

If the player looks at the center of the window, some hexagons will be invisible. Sound plays a crucial role in communicating the players’ progress. For the hexagons an ascending sound was chosen which ends with a “pling” when the hexagon is completely filled. If the player looks away from the center, the sound slows down and is pitched lower. This way, the player can discern the filling speed just by listening to the sound. The teleportation is accompanied by a similar, but lower pitched sound, which ends in a “wush”, and the texts are written letter by letter, with each letter being accompanied with a typewriter-sound. In the WPE, the patients register upon entry by scanning a barcode given to them during their initial visit. The sound of the scanner is used as the sound for activating buttons within the game. Because background noise had an adverse effect on the quality of the audio recording, the gantry rotation, the extension of x-ray panels and the moving of the treatment couch have been implemented with similar sounds taken from an online-library. The implementation of these sounds, the visualization of the gantry room and the incorporation of the protagonist (Radio Robby, WPE-hero) were direct consequences of the requirement evaluation conducted with the patients (Section 3.3, Table 2).

### 4.3. Using the VR game

The patients used the VR application with a Nexus 5 (Google LLC, Mountain View, USA) or Samsung Galaxy S7 (Samsung Electronics Co., Ltd., Suwon, South Korea) smartphone, which use the Android OS, in conjunction with Tera VR glasses. The evaluation of the VR game with the patients was conducted in a hospital examination room.

## 5. Results of the evaluation of the smartphone-based VR game

This section summarizes the feedback from the patients, who tested the VR game (group number 2 in Table 1). First of all, no adverse events, such as nausea or dizziness, were reported in the context of playing the VR game. Irrespective of the patients’ age, everyone showed great interest in using the VR game and the goggles. Each patient played the game to the end. Some used the application quietly and fully concentrated, while others talked while using it and commented what they saw. Some younger patients used it with their parents in the room.

One participant described the world as “like in a video-game where I have to collect things”, “I am in a different world”, and proceeded to answer her mother's question about whether the environment looked cool with “yes” with a giggle. The machines were described as “like imitated”, while one patient who had already completed his therapy mentioned that “the [proton-nozzle] looks different.” The explanation is that the patient was treated in a fixed-beam treatment room and not in a gantry-based treatment room. Each player watched at the animation of the rotating gantry and no one started looking in a different direction. Some mentioned that the sounds were different from the real ones, while others commented that the chosen sounds were “like the real ones.” Moreover, some answered “the sounds” when asked if anything reminded them of the application. The feedback regarding the sounds was the only one where respondents had differing opinions. One patient answered that he was trying to find differences during the treatment to what he remembered from the VR game.

Another element mentioned was the hero, which mostly was named by younger patients, often accompanied by a smile. The background landscape was described as very nice, although one patient mentioned that the actual room would be more appropriate. After describing the problems occurred with that solution, she agreed that the landscape was the better way, too. The teleporting was accepted and understood by all without any problems or questions. Only some of the WPEs representatives had problems with it and did not try it. The button-sound was recognized as the barcode scanner sound by two patients.

The hexagon-games were described as “like honeycombs”, “very nice”, and “the hexagons were top!” The sounds, on the other hand, were criticized by every patient as being squeaky and too loud. They used words like “very annoying”, “like an alarm”, and even after tuning them down a little bit, they still stood out. The first “level” of the hexagon-game was understood by everyone, but some had problems with the second one and did not know what to do. Overall the mini-game was completed with concentration and with a smile.

All but one participant activated the gantry and the nozzle on their own, whilst this one went back and forth between the teleport points of the nozzle and the gantry. For the post evaluation version the texts were worded to avoid this misunderstanding. Almost no one started the last interaction for themselves, and no one took off the goggles, even though the hero requested it in-game. Instructions from the interviewer were needed each time. After lying down, one patient laughingly asked “will I be radiated again now?” Two other patients listed the lying down as the best part of the game and one mentioned “the lying down was cool” as the first comment about the entire VR game.

The application as a whole was described by one patient as “a great idea, very well done, with only a few weaknesses.” Three out of four participants, who were asked for their overall feedback, answered that the VR game did not fall short of their expectations, and no one reported anything as disappointing. The most commonly reported element in need of improvement was the filling sound of the hexagons, which is a small element to fix. One therapy-experienced patient also mentioned that she missed the “blue flashes” in the VR game and wished them to be picked as a theme. Moreover, the idea of giving patients the VR game to use during the 10 days period prior to the treatment, rather than on their second day at the WPE before the break, was very well received by the patients questioned. It was mentioned by one patient before the question was asked.

## 6. Discussion

The goal of the entire project was to find a way to improve the patient's preparation for the first fraction(s) and to test and evaluate this option. The developed VR game was very well received by the patients and staff. Every child, regardless of whether it usually looked up facts for themselves or closed its mind to facts, was eager to test the game and put on the VR goggles. This feedback confirms that VR increases the motivation to use an application.

The elements which were supposed to be recognizable were indeed recognized by every patient, which means that a habituation to the new environment could be possible. The question “do I get irradiated once more now” shows a working connection between the treatment and the VR game. More participants will be needed to conclude an actual habituation. Some children mentioned differences between the real and the virtual world, but according to (33) exploring a new environment rather than fearing it is a good result and leads to positive emotions. Models without any notable differences could even be less beneficial. The free environment compared to a real room was mentioned as more positive than negative. Therefore, even if the modeled room could come with additional benefits, there is no immediate need to change it. The sounds had a similar effect as the models of the machines. They do not exactly match the real sounds, but they are perceived as close to them and were recognized.

For the interaction within the VR game, the gaze-and-wait system worked perfectly. For the mini-games, it would be better to have more possibilities to interact instead of just looking. However, they were still reported as enjoyable. Everyone understood how to interact with objects after a few interactions. There was no need to start interactions with additional buttons or to turn the head while looking at an interactable object. Generally, the VR game was observed with enthusiasm by all participants and described in a very positive way.

It was decided to use the VR game only within our facility instructed by WPE-staff thereby ensuring that the patient does not fall down. Furthermore, a distribution to patients and their families would entail technical support. For instance, only smartphones with a hardware gyroscope facilitate a motion in the virtual world with an acceptable delay.

The limitations of the current study include the lack of a control group and the restriction to qualitative evaluations. For instance, VR acceptability, procedural knowledge and procedural anxiety were not rated as, e.g., in (34, 35). This study focused on the specification, design and implementation of the VR game tailored to the age of the patients and thus, together with the anecdotal evidence demonstrated the proof of concept. Another limitation is that some cancer patients cannot use the developed VR game due to their health conditions, e.g., patients with dizziness or patients which are prone to seizures. No adverse events were reported in this work. This may be explained by the consideration of potential side effects in the design phase (Section 3.5) and in the exclusion criteria (Section 2). Side effects could have been identified with a larger cohort of test patients. For instance, in the study by (36) with a cohort of 61 pediatric cancer patients, three children reported symptoms indicative of simulator sickness after a 10-minute VR experience. In general, side effects reported in VR studies with pediatric patients were mild and infrequent (34). The present work implemented the VR game for a radiation therapy facility manufactured by a commercial proton machine vendor. The renovation of the treatment rooms and upgrades of the treatment machines may entail adaptations of the VR game, which are expected to require little effort. Similarly, the developed VR game could be adapted to particle therapy facilities from other vendors.

The idea is, however, not limited to particle therapy. The workflow and the stress of young patients before the first fraction also pertains to conventional radiation therapy. However, there are differences, e.g., the smaller photon treatment machines. Thus, the requirements for the VR game would have to be re-evaluated for conventional radiation therapy. In this frame, the costs of the roll-out and the maintenance of the VR game and the staff training would be considered. They could outweigh the expected benefit, if the fraction of young patients is rather low as in some conventional radiation therapy departments. Furthermore, hospitals and cancer departments might already utilize room designs optimized for patient comfort, dynamic and customized lighting, video projections, and sounds to improve patient compliance. In this regard, commercial solutions are readily available, e.g., the Philips Ambient Experience (Philips Healthcare, Andover/MA, USA).

Regarding previous studies on immersive VR for radiotherapy patients, a recent review study found that the majority of the VR interventions supporting the treatment of cancer patients pursued a distraction strategy (37). In addition, educational interventions were conducted. For instance (34), reported about a study with pediatric patients, who viewed VR videos of the simulation and the therapy sessions. Recently, seven educational studies using VR in radiotherapy of adult patients were reviewed (20). In addition, VR-based solutions for distraction are commercially available, e.g., Digital Sedation™ (Oncomfort, Wavre/Belgium). The current study aimed to increase the patients’ procedural knowledge and the familiarity with the treatment room in order to reduce the anxiety prior to the first day of treatment. The VR game was tailored to the young target group and the procedures and clinical environment of the proton therapy facility. Therefore, this study extends previous educational studies using immersive VR, which are mentioned above, by combining the educational aspect with an interactive role of the patient through gamification. This approach has been pursed, e.g., in the contex of MRI scans of children (38). To the best of our knowledge, this approach is novel in the context of radiotherapy.

## Data Availability

The datasets presented in this article are not readily available because developed software restricted to the use in our center. Patient interviews are not publically available. Requests to access the datasets should be directed to christian.baeumer@uk-essen.de.
