# Supplementary material for: Development of a smartphone virtual reality game to support the radiation therapy of children and adolescents in proton centers
Source: Front Pediatr. 2023 Jun 20;11:1163022. doi: 10.3389/fped.2023.1163022 (PMC10319066; doi:10.3389/fped.2023.1163022)
Supplement: Supplementary file 1 [file Datasheet1.pdf]

Supplementary Material

1 Structured / prepared questions for the patient interviews

For group 1 and group 2:

Introduction:

- Ask for name, age, hometown. For example, "Hi, how are you? I am ... and you are? (...) How old are you? (...) Where are you from?"
- Settling down in the city of the proton center. E.g. "Have you met other children here? (...) Have you visited nice places?"

Influencing factors during irradiation:

Aim: to obtain a description of the perceptions and the mood during irradiation

The following questions will be asked:

- "How did you feel during the treatment?"

- "How did you perceive the sounds? (general background noises / specific noises)"

- "How was the movement of the table/staff actions in the background?"

Obtain a wish list from the patient for preparation. Afterwards, assess the difference between the expectations/imagination before the first fractions and the actual perceptions. For example, ask,

- "If you had the first treatment in front of you now - what would you like to tell yourself?"

Acceptance of the medium and content:

Assess previous experience with the medium:

"What do you think about computer / mobile games?"

"Which games / genres? - Do you have a favorite game?"

Assess experience with / opinion about virtual reality:

- Have you ever heard of virtual reality? (...)
- [give a short explanation if necessary]
- Ask for opinion about virtual reality

Opinion about content:

- “What do you think about comics and superheroes? Have you met Radio-Robby? Who is your favorite superhero?”
- “Do you read a lot? Do you read long texts? What is important to you in what you read?”

General requests:

“What else is on your mind?”

For group 2, above questions were followed by a free interview about the patient’s experience with the VR-game.
